# Management of a failed endodontic treatment for a maxillary second molar with two separate palatal roots

**DOI:** 10.1002/ccr3.1708

**Published:** 2018-07-13

**Authors:** Ahmed Al Qahtani, Saleem Abdulrab, Hatem Alhadainy

**Affiliations:** ^1^ Al Yamamah Hospital Ministry of Health Riyadh Saudi Arabia; ^2^ Department of Restorative Dental Science Alfarabi Dental College Riyadh Saudi Arabia; ^3^ Department of Dentistry Faculty of Medicine and Dentistry Alberta University Edmonton AB Canada; ^4^ Department of Endodontics College of Dentistry University of Tanta Tanta Egypt

**Keywords:** anatomical variations, maxillary second molar, palatal roots, retreatment

## Abstract

Complexity of root canal system and variations in internal anatomy of teeth require careful analysis of preoperative cone beam computed tomography or multiangle radiographs to locate and identify possible extra roots or canals. A fourth canal in upper molars is expected, and much effort should be made when planning the endodontic treatment to avoid missing a canal.

## 1 INTRODUCTION

One of the most challenging goals for endodontists is managing the complexity of the root canal system. Despite all procedural protocols, missing a canal or an additional root can lead to treatment failure and poor prognosis. A second maxillary molar with a second palatal root is rare. This case report introduces a management for a failed treatment of distinct palatal roots in a second maxillary molar.

The anatomical complexities of the root canal system have been accentuated as one of challenges for endodontists and researchers. Despite all procedural protocols, missing a canal or an extra root can lead to treatment failure. Permanent maxillary molars often have three roots, one palatal and two buccal with a root canal in each root. However, a second mesiobuccal canal is often present, and thus, a tooth having four canals is common. Distobuccal and palatal canal usually have one canal each, although on rare occasions, either may have an extra canal. Extra root may also present in seldom conditions as fused or separate root.^1^, ^2^ Stone and Stroner 3 reported approximately 2% of maxillary molars with more than one palatal canal when examined 500 extracted molars, while Libfeld and Rotstein 4 found only 0.4% incidence of extra palatal canal in 200 upper second molars and 1000 radiographs. Christie et al^5^ indicated that maxillary second molar has highest incidence of extra palatal canals in double palatal roots. Peikoff et al^6^ reported that the incidence of four separate roots with four separate canals is 1.4% in maxillary second molars.

Several studies 7, 8, 9, 10, 11, 12, 13 confirmed the morphological variations in the root canal system in the palatal root of the maxillary second molar. Christie et al^5^ proposed a classification for four‐rooted maxillary second molars into three types. In type I, the two palatal roots are often longer and more tortuous and divergent than buccal roots that are less divergent with a “cow‐horn” shape. The two palatal roots in type II are often shorter than type I with blunt apices and run almost parallel to each other. The palatal roots in type III are less divergent and often shorter than buccal roots. Analysis of high‐resolution microcomputed tomography (micro‐CT) resulted in a modified classification of four‐rooted maxillary molars.^7^ This modification is similar to Christie et al^5^ with the addition of type IV that describes maxillary molars with three buccal roots.

It is sometimes difficult to clinically detect a double palatal root because the second root canal may be superimposed by buccal root canals. Subsequently, root canal treatment becomes complicated and the failure rate is increased. The current report presents management of failed endodontic treatment of a maxillary second molar with two palatal root canals.

## 2 CASE REPORT

**Figure 1:**
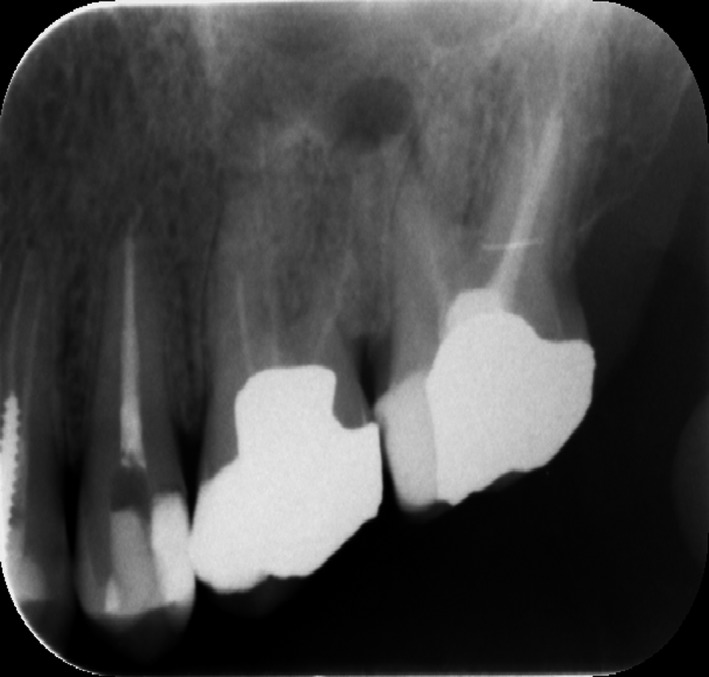
Teeth no: 26 and 27 Showed a Failed Endodontic Treatment with Poor Obturation and Missing Untreated Canals

A 35‐year‐old male patient presented to the East Riyadh dental center in Riyadh city, Saudi Arabia. He was referred from a prosthodontic clinic to correct his previous treatment. The patient was in good health with no apparent systemic disease. On clinical examination, the teeth 26 and 27 were asymptomatic with large previous restorations in both teeth. A preoperative periapical radiograph revealed the presence of four roots in tooth 27. This tooth had previous endodontic treatment with poor obturation and missing untreated canals (Figure 1).

**Figure 2:**
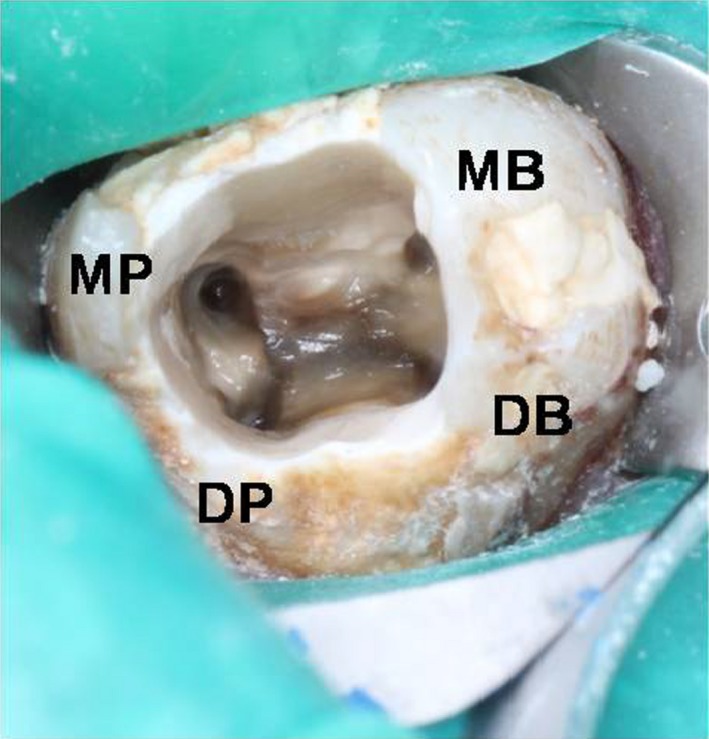
Access Cavity of Tooth no 27 Showed Rhomboidal Outline with Four Canal Orifices

A diagnosis of asymptomatic apical periodontitis was made, and nonsurgical retreatment for teeth 26 and 27 was planned. The old restoration of tooth 27 was removed following local anesthesia and rubber dam application. Rhomboidal outlined access cavity was then prepared to obtain a straight‐line access to all canals. There were four canal orifices, two buccal and two palatal canals located on the floor of the pulp chamber (Figure 2).

**Figure 3:**
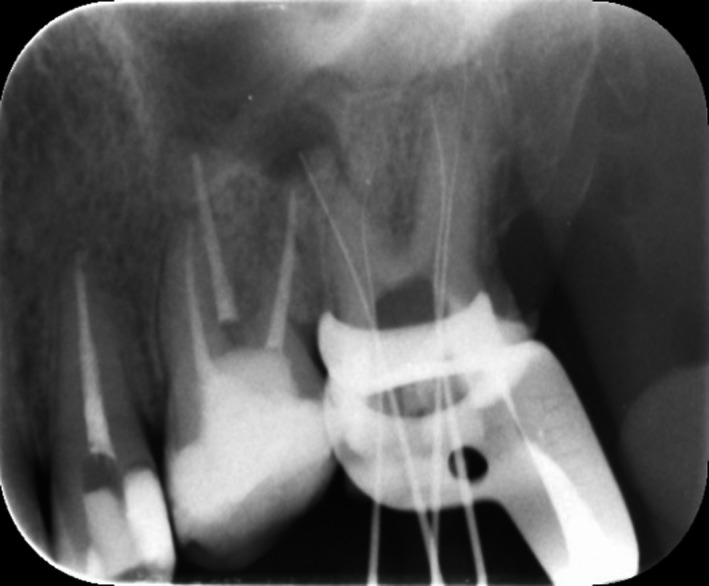
Working Length Determination of Tooth no 27

Old gutta‐percha filling was removed using a Gates Glidden drills and H file (Mani, Inc., Japan) and chloroform as solvent. Working length was determined by the apex locator (Root ZX, J. Morita Corp., Tokyo, Japan) and confirmed radiographically (Figure 3). Canal preparation was performed using ProTaper Universal (Dentsply‐maillefer, Ballaigues Germany) files up to F3 in mesiopalatal and in F4 distopalatal by while other canals prepared up to F2. The canal was irrigated between each instrument with 5.25% NaOCl, and final irrigation was done using 17% EDTA (Meta Biomed Co. Ltd., Cheongju City, Chungbuk, Korea). Patency was kept by recapitulation with a No.10 file.

**Figure 4:**
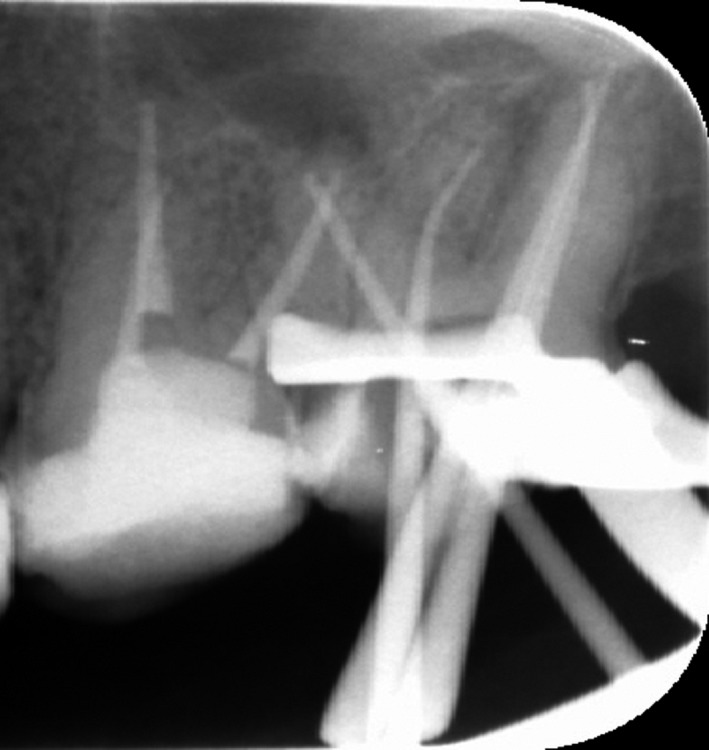
Master Apical Cone Radiograph

**Figure 5:**
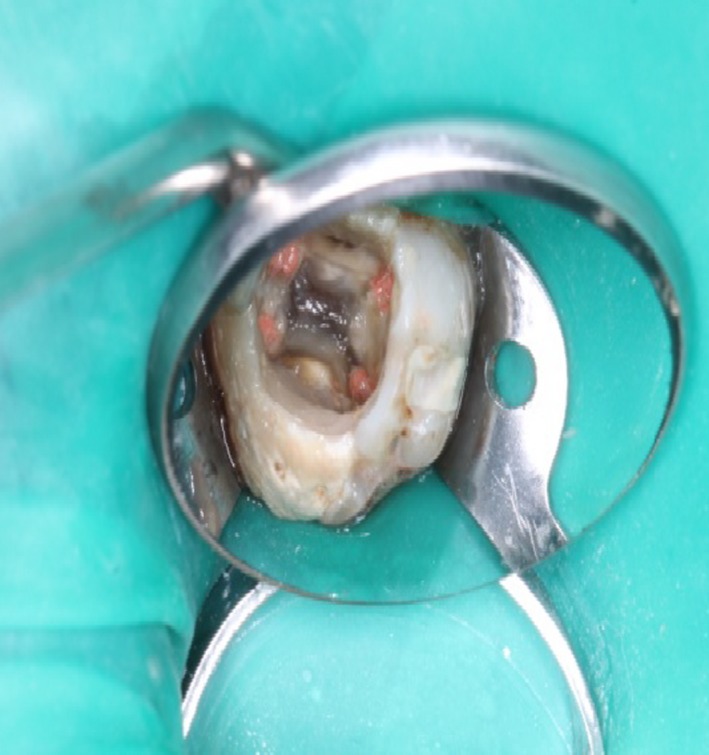
Access Cavity After Obturation Showed Canals Orifice Sealed with Gutta‐Percha

**Figure 6:**
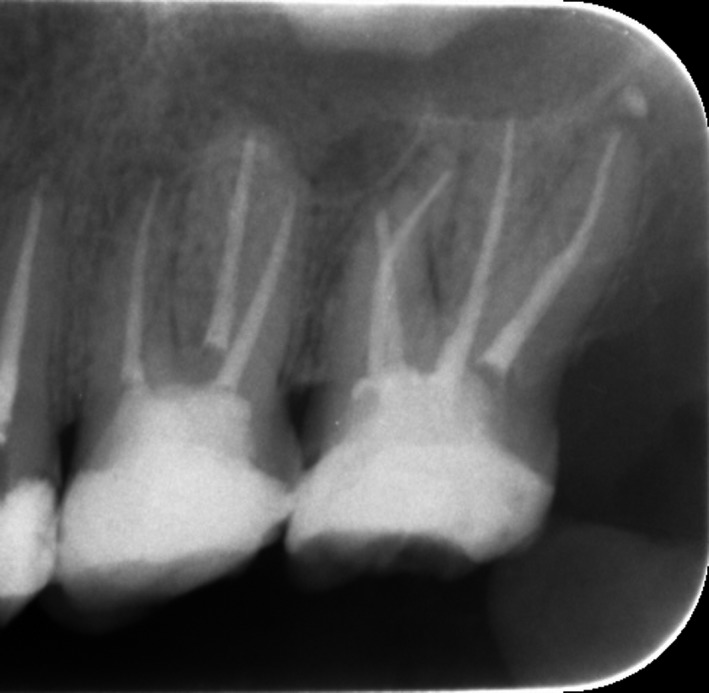
Postoperative Radiograph Showed Obturation of Both Teeth

A master cone radiograph was taken (Figure 4), and canals were obturated with gutta‐percha and AH plus sealer (Dentsply, Maillefer, Germany). Gutta‐percha was filled using continuous wave of condensation technique with a system B heat source (SybronEndo, Orange, CA, USA), and a cordless obturation gun with gutta‐percha pellets (Meta Biomed Co. Ltd.) was used for back fill (Figure 5). The access cavity was sealed with a temporary filling material (Cavit^™^ G, 3M ESPE, Germany), postoperative radiographs were taken (Figure 6), and the patient was then referred back to the prosthodontist. Tooth #26 was retreatment as the same manner as #27. We tried to locate the MB2 by careful examination of the pulpal floor under dental operating microscope and trephination using ultrasonic tip but we could not find it.

## 3 RESTORATION

**Figure 7:**
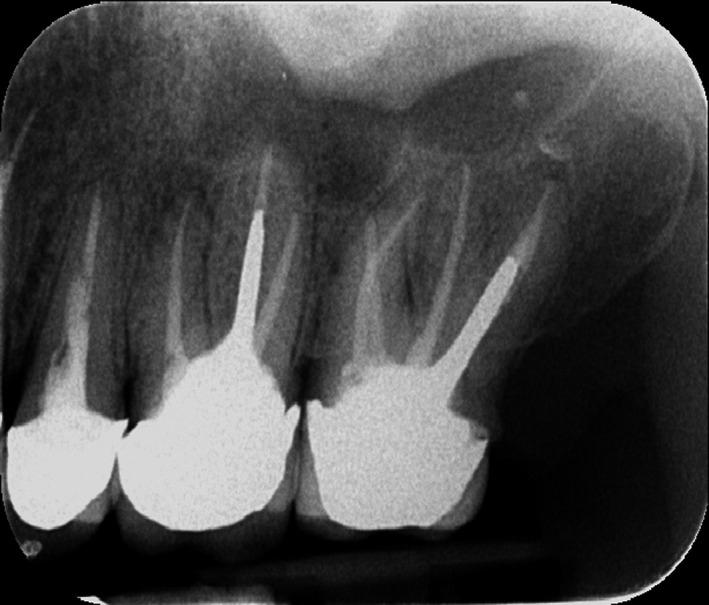
Postoperative Radiograph after Place Cast Post And Coronal Restoration Using Zirconia Crown

On completion of the root canal therapy, a post space was created in the largest canal, which is the distopalatal canal, using a post drill kit (Relyx 3M ESPE). The remaining tooth structures were not sufficient to hold a coronal filling; therefore, the tooth was restored using a cast post and core that was covered with zirconia crown. A periapical radiograph was taken (Figure 7). On follow‐up, the patient was asymptomatic at the 1‐month and 6‐month recall appointments.

## 4 DISCUSSION

The second maxillary molar has a complex root canal system, and one of the reasons for failure of endodontic treatment is because the entire root canal system was not located and cleaned.^8^ The incidence of two palatal roots or two palatal root canals in second maxillary molars is rare and reported to be approximately 0.4%‐2%.^3^, ^9^, ^10^

Christie et al^5^ proposed a classification for four‐rooted maxillary second molars into three types. The present case is type 1 according to this classification. When two palatal roots exist in maxillary molars, one of them is the normal palatal root and the other is a supernumerary structure that can be located either mesial (radix mesiolingualis) or distal (radix distolingualis) of the palatal root.^11^

Careful observation of preoperative radiographs is required to diagnose morphological variations. Failure to diagnose these variations is usually due to superimposition of anatomic structures on palatal canals of maxillary molars.^10^ Thus, radiographs should be taken at different angles to detect and locate the roots. Using magnification such as loupes, surgical microscopes, and sodium hypochlorite bubbling may be helpful in locating and better visualizing anatomic aberrations.

In a recent review on root anatomy and canal configuration of maxillary second molars,^14^ the main reported cases were related to the palatal root, most of which involved reporting the presence of two separate palatal roots. The three‐rooted anatomy was most common, while the four‐rooted anatomy had the lowest prevalence. The main method of anatomical investigation in case reports was periapical radiography, and the main method in morphological studies was the CBCT technique.

In summary, this report involves successful retreatment of a maxillary second molar with an additional palatal root. Careful analysis of the preoperative radiograph and examination of the floor of the pulp chamber is required to detect this anatomical variation. This is usually facilitated by an alteration in access cavity shape to a rhomboidal one.

## 5 CONCLUSION

Knowledge and awareness of internal anatomical variations of teeth and careful analysis of preoperative multiangle radiographs are important to locate and identify extra roots and all canals. Neglecting this anatomical variation leads to failure of treatment.

## AUTHOR CONTRIBUTIONS

AAQ: involved in conception, materials, funding. SA: wrote the article and involved in study design. HA: contributed in the study design and article writing.

## CONFLICT OF INTEREST

The authors declare no conflict of interests for the publication of this article.
